# Cerebrospinal Fluid Immunoglobulin Kappa Light Chain in Clinically Isolated Syndrome and Multiple Sclerosis

**DOI:** 10.1371/journal.pone.0088680

**Published:** 2014-04-02

**Authors:** Makbule Senel, Hayrettin Tumani, Florian Lauda, Stefan Presslauer, Rehaneh Mojib-Yezdani, Markus Otto, Johannes Brettschneider

**Affiliations:** 1 Department of Neurology, University of Ulm, Ulm, Germany; 2 Department of Neurology, Wilhelminenspital, Vienna, Austria; Innsbruck Medical University, Austria

## Abstract

**Background:**

Oligoclonal bands (OCB) are the most widely used CSF test to support the diagnosis of MS and to predict conversion of clinically isolated syndrome (CIS) to multiple sclerosis (MS). Since OCB tests are based on non-quantitative and difficult to standardise techniques, measurement of immunoglobulin kappa free light chains (KFLC) may represent an easier to use quantitative test.

**Methods:**

KFLC were measured in CSF and serum of 211 patients using ELISA. These include patients without any inflammatory central nervous system reaction (NIND, n = 77), MS (n = 20), viral CNS infections (V-CNS-I, n = 10), neuroborreliosis (NB, n = 17) and other bacterial CNS infections (B-CNS-I, n = 10). Furthermore a cohort of 77 patients with CIS, including 39 patients that remained CIS over follow-up of two years (CIS-CIS) and 38 patients that developed MS over the same follow-up time (CIS-MS).

**Results:**

CSF-serum ratio of KFLC (Q KFLC) was elevated in all patients with MS, 86.8% of patients with CIS-MS and 61.5% of patients with CIS-CIS. It was significantly elevated in CIS with presence of OCB (p<0.001). Q KFLC significantly correlated with other CSF variables such as CSF leukocyte count (p<0.001, R = 0.46), CSF CXCL13 levels (p<0.001, R = 0.64) and also intrathecal IgG synthesis (p<0.001, R = 0.74) as determined by nephelometry and quotient diagram. OCB were detected in 66.7% of CIS-CIS and in 92.1% of CIS-MS.

**Conclusions:**

Although the measurement of CSF KFLC is a rapid and quantitative easy to standardize tool, it is almost equal but not superior to OCB with regard to diagnostic sensitivity and specificity in patients with early MS.

## Introduction

In most patients who develop multiple sclerosis (MS), the disease initially presents with a first relapse-like episode known as clinically isolated syndrome (CIS) [1]. Occasionally, the disease is incidentally detected in magnetic resonance imaging (MRI) through asymptomatic lesions suggestive of MS as radiologically isolated syndrome (RIS) [2]. Given the importance of an early treatment of MS with disease-modifying immunomodulatory therapies that are more effective in early stages of disease [3], the clinical challenge in CIS or RIS is to identify patients with a high risk of future relapses that could be associated with debilitating neurological deficits. Consequently, an abundance of neuroimaging and biochemical markers have been evaluated as possible predictors of future relapses in CIS and early MS [4]–[9]. Cerebrospinal fluid (CSF) is a promising source of biochemical markers in MS, since it is the body fluid with the closest anatomical contact to MS pathology, and could reflect biochemical changes associated with the disease [10], [11]. So far, immunoglobulin G (IgG) oligoclonal bands (OCB) are the most widely used CSF test to predict MS [9], [11], [12]. However, determination of OCB using isoelectric focusing (IEF) on gels followed by immunoblotting demands considerable methodological experience and is both labour-intensive and difficult to standardise [13].

Several studies indicated that elevated immunoglobulin kappa free light chains (KFLC) and lambda free light chains (LFLC) in the CSF may offer a quantitative tool to support the diagnosis of MS [14]–[20]. However, most previous studies focused on MS, cohorts of CIS were usually small, and prospective data was scarce.

Here, we provide 1) CSF reference values for KFLC based on a highly sensitive ELISA to evaluate the relevance in MS, CIS and pathogen-related CNS diseases and 2) a systematical analysis of the prognostic relevance of KFLC regarding the occurrence of further relapses in a large and clinically well-defined cohort of patients with CIS. We compare the prognostic relevance of KFLC in CIS to MRI Barkhof criteria [21] and markers reflecting the polyspecific intrathecal B-cell response, including OCB [22] and intrathecal IgG synthesis.

## Methods

### Patients

211-paired CSF and serum samples from the Department of Neurology, University of Ulm (Germany) were analysed. These included 77 patients with CIS collected in a prospective study with a follow-up time of two years, as previously described [23], including 39 patients that remained CIS over a follow-up (CIS-CIS) and 38 patients that developed MS of the relapsing-remitting subtype (CIS-MS) over the same period (Table 1). We furthermore included 20 patients with MS according to modified McDonald criteria [26]. Disability was rated using Kurtzke's Expanded Disability Status Scale (EDSS) [27] by two experienced neurologists (HT, FL) unaware of any results on the CSF biomarkers. Lumbar puncture was performed as part of the routine diagnostic work up using an atraumatic 22G Sprotte needle and prior to application of steroids in all patients. All samples were handled and stored in accordance with BioMS guidelines [28].

**Table 1:** Demographic data and basic cerebrospinal fluid findings.

	N (Male/Female)	Age (Years)	CSF cell count (/µl)	CSF protein (mg/l)	Qalb (×0.001)	CSF lactate (mmol/l)	OCB (%)
MS	20 (7/13)	35 (30–40)	7 (2–12)	490 (462–578)	5.85 (5.05–7.05)	1.7 (1.5–1.8)	18 of 20 (90.00)
CIS	77 (32/45)	34 (24–43)	4 (2–11)	409 (345–549)	5.1 (3.9–6.3)	1.6 (1.4–1.8)	61 of 77 (79.22)
B-CNS-I	10 (5/5)	61 (39–72)	1980 (107–3100)	3425 (1200–4300)	58.1 (38.2–90.2)	7.9 (3.1–11.1)	1 of 10 (10.00)
NB	17 (11/6)	58 (31–68)	80 (48–184)	1080 (859–1430)	13.6 (9.6–17.1)	2 (1.6–2)	14 of 17 (82.35)
V-CNS-I	10 (9/1)	44 (26–63)	176 (54–263)	794 (684–1412)	11.75 (9.3–21.8)	2 (1.6–3)	2 of 10 (20.00)
NIND	77 (44/33)	54 (44–67)	1 (1–2)	576 (406–765)	7.1 (5–10.4)	1.8 (1.6–2.1)	0 of 77 (0)

Data are shown as the median and IQR. Abbreviations: B-CNS-I bacterial central nervous system infections, CIS clinically isolated syndrome, CSF cerebrospinal fluid, MS multiple sclerosis, NB neuroborreliosis, NIND non-inflammatory neurological diseases, OCB oligoclonal IgG bands, V-CNS-I viral central nervous system infections.

The inflammatory control groups consisted of 17 patients with neuroborreliosis (NB) (according to criteria by Kaiser [29]), 10 patients with other bacterial CNS infections (B-CNS-I, including meningitis or meningoencephalitis caused by Mycobacterium tuberculosis, Treponema pallidum, Listeria monocytogenes, Haemophilus influenzae, Staphylococcus aureus, Streptococcus pneumoniae or other Streptococci), and 10 patients with viral CNS infections (V-CNS-I, including meningitis or meningoencephalitis caused by herpes simplex virus (HSV), varicella zoster virus (VZV), Epstein-Barr virus (EBV), tick- borne encephalitis Virus (TBEV) and others). The normal controls showed no symptoms in neurological examination, unremarkable routine CSF findings, as well as no evidence of a structural, haemorrhagic or inflammatory lesion in MRI (non-inflammatory neurological diseases (NIND)) (Table 1).

### Ethics statement

Written informed consent was obtained from all patients in accordance with the Declaration of Helsinki, and the study was approved by the ethics committee of the University of Ulm (No. 282/08).

### Determination of MRZR, CXCL13, and OCB

MRZR (Measles, Rubella, Zoster Reaction) was determined as previously described (23) using an enzyme-linked immunosorbent assay (ELISA) according to the instructions as supplied by the manufacturer (Genzyme Virotech, Rüsselsheim, Germany). Quantitative expression of the intrathecal immune response was based on calculation of the CSF/serum quotients (Q) of specific antiviral IgG antibodies, and the intrathecal synthesis of antibodies was detected by calculation of the corresponding antibody indices (AI). AI values ≥1.5 were considered to be indicative of intrathecal IgG synthesis against the respective antigen, and MRZR was considered positive if two or more AI values were ≥1.5.

CXCL13 was measured using ELISA (Quantikine; R&D Systems, Minneapolis, MN) according to the instructions as supplied by the manufacturer. Samples of 50 ml CSF and 50 ml serum were used for the ELISA. Details on the application of the assay for CSF have been published previously by our group [24], [30].

CSF leukocyte count (cells/cu.mm), total protein (g/L), lactate (mmol/L), the albumin CSF/serum concentration ratio (Qalb), immunoglobulins G, A and M were obtained as previously described [31], [32].

OCB were detected by isoelectric focusing (IEF) on agarose gels followed by immunoblotting using an IgG-specific antibody staining. Paired CSF and serum adjusted for protein concentrations were applied in the same assay as described earlier [33].

### ELISA for determination of KFLC and LFLC

Immunoglobulin Kappa and Lambda Free Light Chains (KFLC and LFLC) were measured by quantitative enzyme-linked immunosorbent assay (ELISA) (BioVendor) according to the instructions supplied by the manufacturer. All samples were stored at −80°C until analysis according to the sampling and storage protocol of BioMS [28]. For validating and assessing assay accuracy for CSF, spike-and-recovery experiments were performed. ELISA matrix recovery was acceptable, with an average recovery of 103.4% (range 99.1–108.8%, n = 3) for CSF-KFLC and 95.0% (range 88.4–104.5%, n = 4) for CSF-LFLC. The standards of the ELISA were prepared as recommended by the provider. Samples and standards are incubated in microplate wells pre-coated with monoclonal anti-human immunoglobulin KFLC antibody. The lower detection limit for KFLC was 6 µg/l. The inter-assay CV was 6.17% for a lower KFLC control (n = 8) and 2.52% for a higher KFLC control (n = 8), which were in range with the inter-assay CV indicated by the provider. Serum samples were diluted 200 fold (for both KFLC and LFLC). CSF samples exceeding KFLC level of 320 µg/l or LFLC level of 560 µg/l were remeasured with 2–40 fold dilutions.

### MRI Analysis

MRI scans of the brain and spinal cord were performed on a 1.5 tesla whole-body MRI (Symphony Siemens, Erlangen, Germany) according to a previously fixed protocol including T1-weighted spin-echo (SE) axial slices with and without application of gadolinium-DTPA as well as T2-weighted SE axial slices. Barkhof criteria [21] were applied as diagnostic criteria.

### Statistical Analysis

Statistical analysis was performed using SPSS (version 19.0; SPSS Inc., Chicago, IL, USA). Differences of FLC between groups were analyzed using Kruskal-Wallis test followed by single pairwise comparisons (Mann-Whitney U-test). The Correlation was analysed using Spearman rank order correlation. To test for normality, the Kolmogorov-Smirnov-test was applied. Sensitivity was calculated as (true-positive/[true-positive+false-negative]), specificity was calculated as (true-negative/[true-negative+false-positive]). The positive predictive value (PPV) was calculated as (true-positive/[true-positive+false-positive]), and the negative predictive value (NPV) as (true-negative/[true-negative+false-negative]). For all diagnostic values the exact 95% confidence intervals were given [34]. P-values below 0.05 were considered to be significant.

## Results

### KFLC in CSF and serum

Patients with MS and CIS showed significantly elevated CSF KFLC levels as compared with NIND (p<0.001) (Table 2). CSF KFLC levels were also elevated in cases of viral and bacterial CNS infections (B-CNS-I and V-CNS-I), especially in cases of NB (p<0.001, as compared with NIND). In contrast, serum KFLC was significantly lower in cases of MS (p = 0.001) and CIS (p<0.001) as compared with NIND (Table 2). Patients with MS showed significantly elevated CSF-serum ratio of KFLC (Q KFLC) as compared with CIS (p = 0.004), B-CNS-I (p = 0.022), V-CNS-I (p = 0.001) and NIND (p<0.001). Q KFLC in MS was also higher as compared with NB, though this did not reach statistical significance (p = 0.821).

**Table 2:** KFLC in CSF and serum.

	CSF KFLC (µg/l)	Serum KFLC (µg/l)	Q KFLC (×0.001)
MS	615.0 (377.9–1340.9)	7762.3 (5246.0–9477.3)	113.2 (46.4–192.8)
CIS	125.5 (14.2–723.6)	7035.0 (4560.4–8992.6)	14.9 (2.8–115.6)
B-CNS-I	403.9 (231.0–748.9)	8829.5 (6011.8–14721.6)	41.5 (26.1–73.5)
NB	1503.3 (641.2–2241.2)	10524.0 (5848.6–15627.4)	97.5 (42.8–279.7)
V-CNS-I	144.2 (88.7–396.6)	8970.6 (5228.4–14266.2)	19.3 (11.5–32.6)
NIND	13.1 (4.0–25.8)	11045.4 (7648.0–14564.2)	1.1 (0.1–1.7)

Immunoglobulin kappa free light chain (KFLC) in CSF and serum of patients with MS, CIS, pathogen-related diseases and NIND (for abbreviations see legend of table 1). Q KFLC: CSF- serum ratio of KFLC. Data are shown as the median and IQR.

### CSF reference values for KFLC

**Figure 1:**
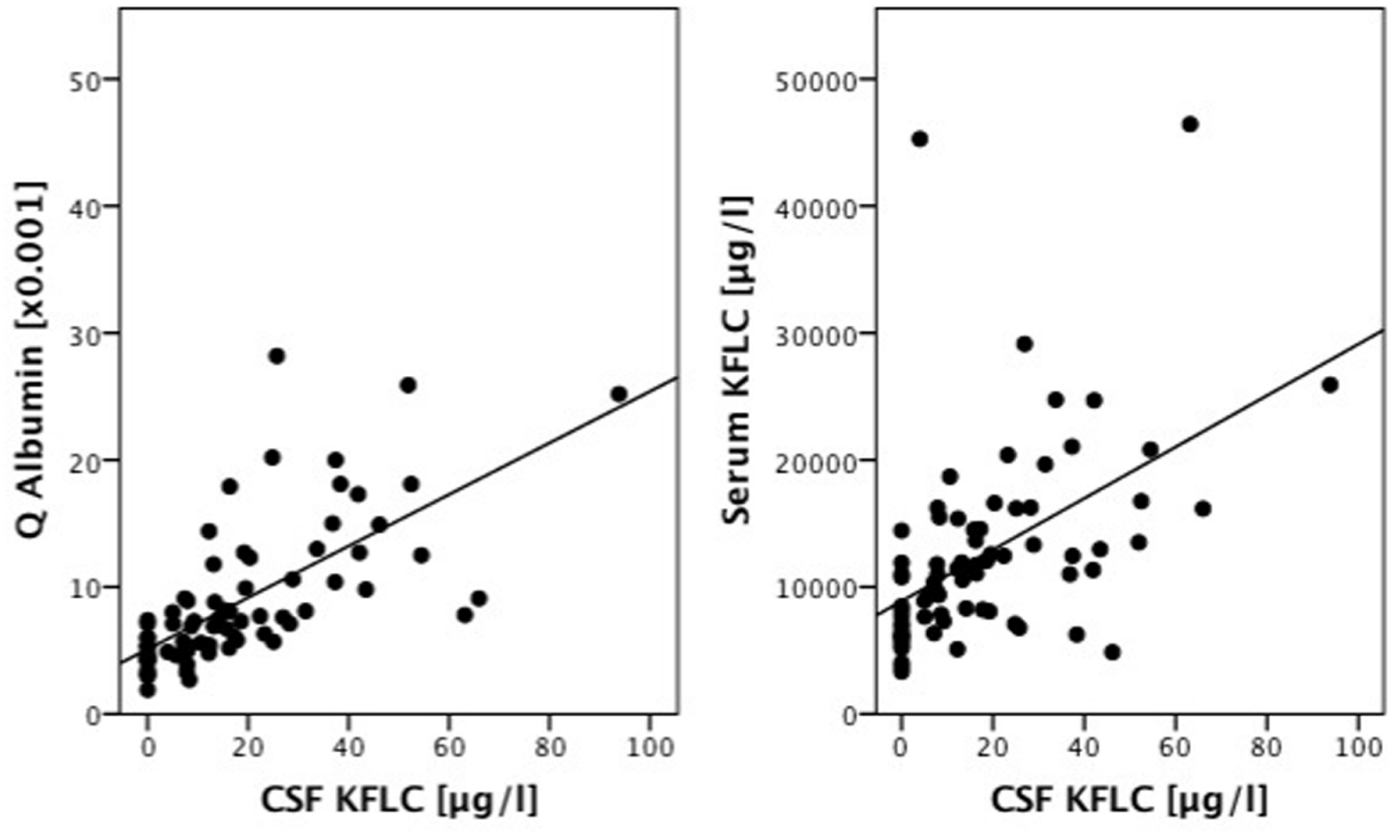
Correlation among cerebrospinal fluid kappa free light chain (CSF KFLC) levels in non-inflammatory neurologic diseases (n = 77) and CSF-serum ratio of albumin (Q Albumin)(left; p<0.001, R = 0.775) and serum KFLC levels (right; p<0.001, R = 0.587) are shown. The straight lines are regression lines.

**Figure 2:**
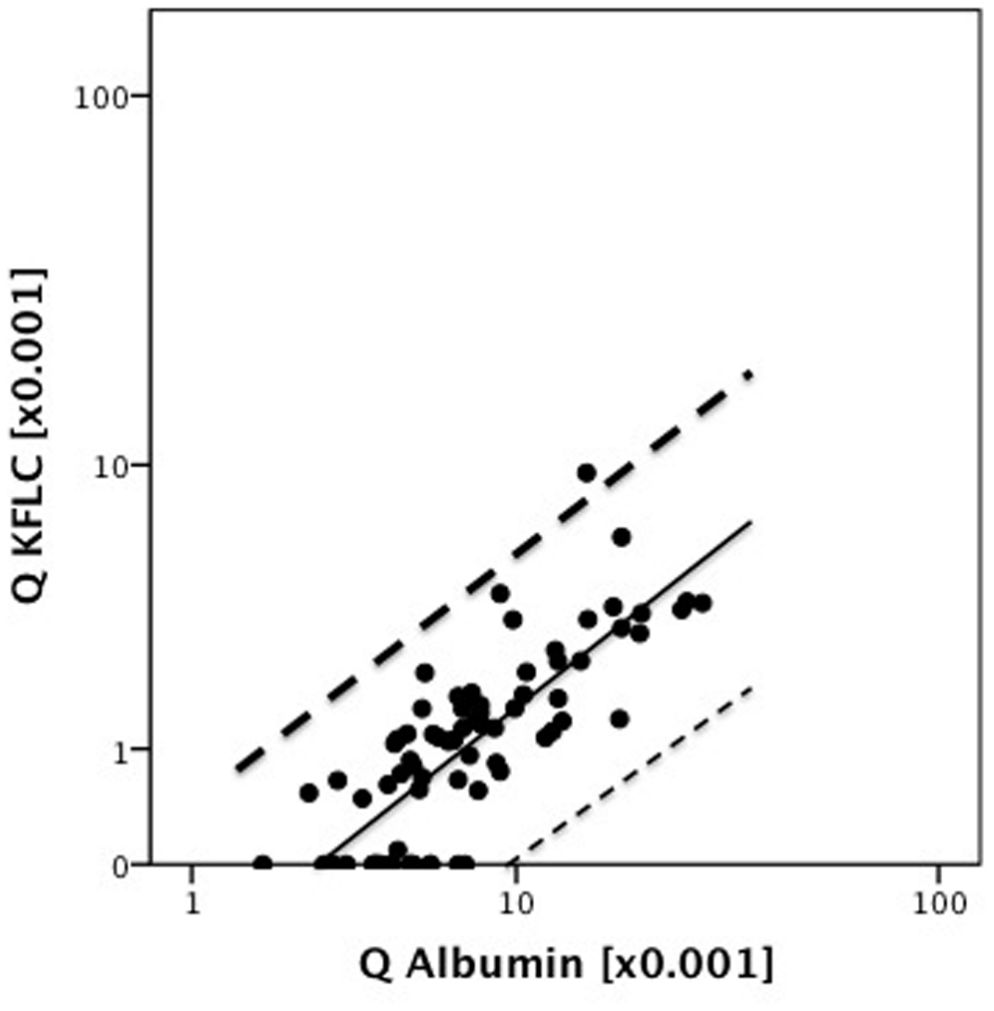
Cerebrospinal fluid – serum ratio of kappa free light chain (Q KFLC) is plotted against CSF-serum ratio of albumin. Dashed lines indicate the 99% confidence interval of the linear regression line (straight line). The upper 99% confidence interval (dark dashed line) indicate the approximately upper reference value of Q KFLC based on a control group of 77 non-inflammatory neurologic diseases and a range of Q Albumin from 1.9 to 28.2.

In NIND, CSF KFLC correlated significantly with serum KFLC (p<0.001, R = 0.587) and also significantly with CSF-serum ratio of albumin (Q albumin) (p<0.001, R = 0.775) (Figure 1). Therefore we plotted CSF-serum ratios of KFLC (Q KFLC) versus the respective ratios of albumin and indicate the upper 99% confidence interval of the linear fit line as the approximately upper reference value of Q KFLC (Figure 2).

### KFLC and oligoclonal IgG bands

**Figure 3:**
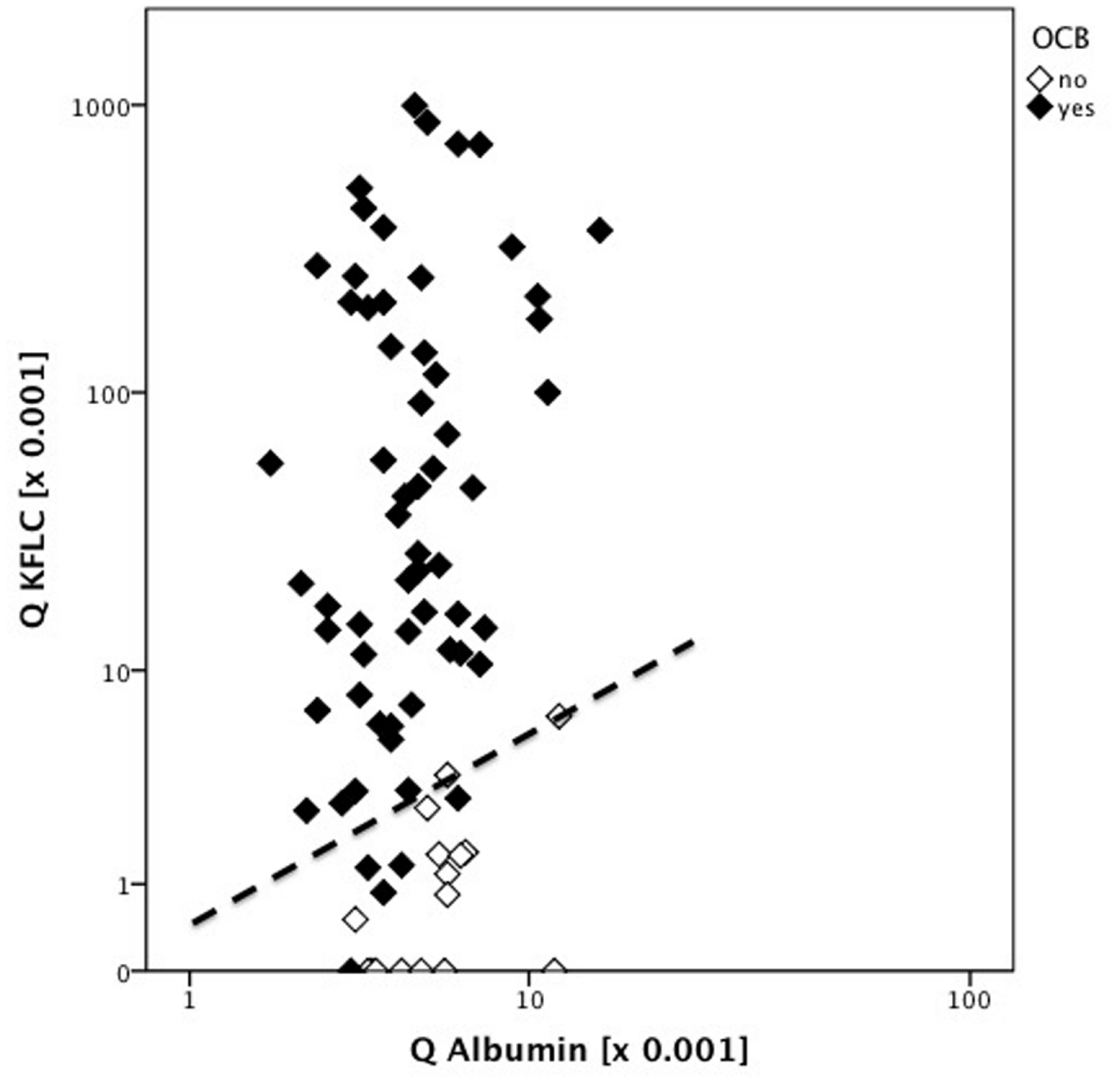
CSF-serum ratio of KFLC (Q KFLC) was elevated in patients with positive oligoclonal IgG (OCB). Q KFLC of 77 CIS patients are shown. Dashed line indicates the approximately upper reference value of Q KFLC, described in figure 2. Black rhomb indicate positive OCBs, white rhomb indicate negative OCBs.

**Figure 4:**
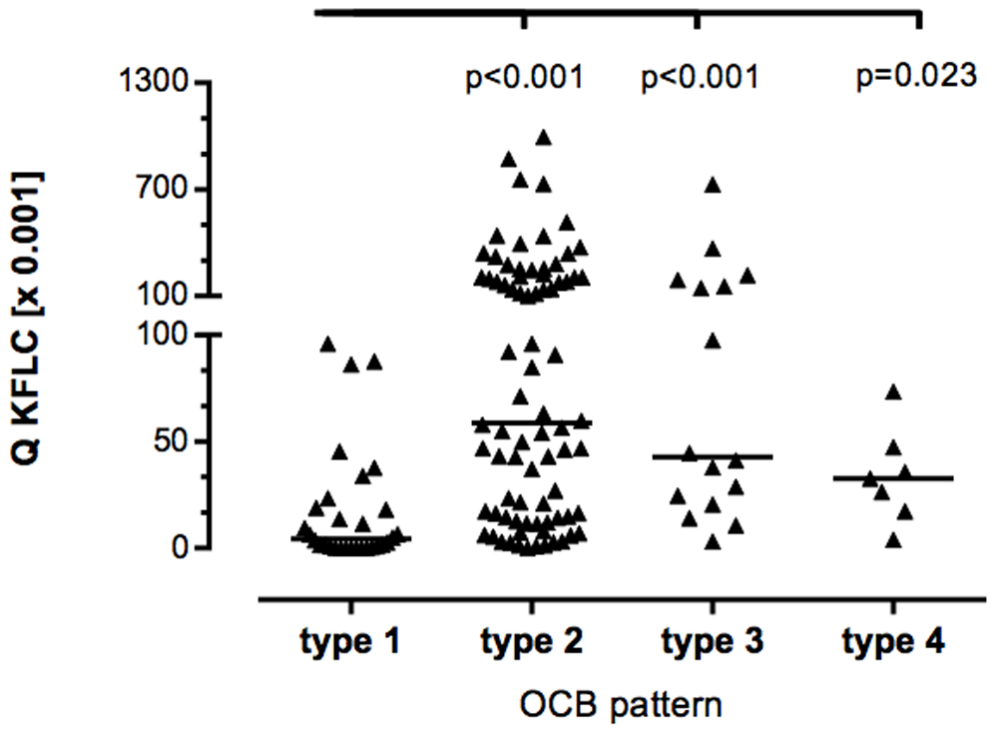
CSF-serum ratio of KFLC (Q KFLC) among different oligoclonal IgG band patterns. There are five classic patterns of oligoclonal bands [48] (type 1, no bands in CSF and serum; type 2, oligoclonal IgG bands in CSF but not in serum, indicative of intrathecal IgG synthesis; type 3, oligoclonal bands in CSF plus identical oligoclonal bands in serum and CSF, indicative of intrathecal IgG synthesis; type 4, identical pattern of oligoclonal bands in CSF and serum. There was no patient with type 5 (identical patterns of monoclonal bands in CSF and serum) in the study. Horizontal solid line indicates median, Kruskal-Wallis test among groups revealed a significant difference (p<0.001), significant P-values for pairwise comparisons (Mann-Whitney U test) are displayed.

Q KFLC was significantly elevated in cases with presence of CSF oligoclonal IgG bands (OCB) (p<0,001) (Figure 3). Q KFLC was significantly elevated in patients with OCB type 2 and 3 patterns, that are indicative for intrathecal IgG synthesis, as compared with type 1 (i.e. no bands in CSF and serum) (p<0.001, Figure 4). In cases of type 4, i.e. identical oligoclonal bands in CSF and serum, indicative of a systemic but not intrathecal immune reaction, Q KFLC was also significantly higher as compared with type 1 (p = 0.023, Figure 4).

### KFLC in MS and CIS

In cases of CIS, Q KFLC was positive (i.e. above the discrimination line described in figure 2) in 61.5% of patients with CIS-CIS (24 of 39 patients with CIS that remained CIS over a follow-up of two years) and in 86.8% of patients with CIS-MS (33 of 38 patients with CIS that developed MS over a follow-up of two years). OCB were detected in 66.7% of CIS-CIS and in 92.1% of CIS-MS. Intrathecal IgG synthesis (according to quotient diagrams) were detected in 35.9% of CIS-CIS and 43.2% of CIS-MS.

In patients with CIS, Q KFLC showed a significant correlation with CSF leukocyte count (p<0.001, R = 0.46), CSF CXCL13 levels (p<0.001, R = 0.64) and also intrathecal IgG synthesis (p<0.001, R = 0.74). Q KFLC was significantly higher in CIS with positive OCB as compared to CIS without OCB (p<0.001, Figure 3). Similarly, Q KFLC was significantly higher in CIS with positive MRZR as compared to CIS without MRZR (p<0.001). CIS patients with EDSS > = 2,5 at diagnostic lumbar puncture showed higher levels of Q KFLC as compared to patients with EDSS <2,5 (p = 0.03). In contrast, Q KFLC correlated neither with duration of symptoms (p = 0.06, R = 0.22) nor with gadolinium-enhancing lesions in MRI (p = 0.09).

All patients with MS showed elevated Q KFLC, 90% of them showed positive OCBs.

### Predictive value of KFLC in CIS

Of all markers investigated, OCB showed the highest sensitivity for conversion of CIS to MS (92.1%), which could not be improved by adding any of the other parameters (Table 3). KFLC were nearly as sensitive as OCB (86.8%), and both showed a low specificity (33.3% and 38.4%, respectively). In contrast, Barkhof criteria showed the highest specificity (88.2%).

**Table 3:** Sensitivity, specificity, positive and negative predictive value for elevated KFLC, MRI parameters and OCB regarding conversion of clinically isolate syndrome to definite multiple sclerosis.

	N	Sensitivity (%)	Specificity (%)	PPV (%)	NPV (%)
Q KFLC	77	86.8 (71.9–95.6)	38.5 (23.4–55.4)	57.9 (44.1–70.9)	75.0 (50.9–91.3)
OCB	77	92.1 (78.6–98.3)	33.3 (19.1–50.2)	57.4 (44.1–70.0)	81.3 (54.4–96.0)
Intrathecal IgG-Synthesis	76	43.2 (27.1–60.5)	64.1 (47.2–78.8)	53.3 (34.3–71.7)	54.3 (39.0–69.1)
IgG-Index >0.70	76	43.2 (27.1–60.5)	64.1 (47.2–78.8)	53.3 (34.3–71.7)	54.3 (39.0–69.1)
Barkhof	66	12.5 (3.5–29.0)	88.2 (72.5–96.7)	50.0 (15.7–84.3)	51.7 (38.2–65.0)

Data are shown as percent and 95% confidence interval. Q KFLC = CSF-serum ratio of KFLC above the approximately upper reference value described in figure 2. OCB = cerebrospinal fluid oligoclonal bands of IgG class not detectable in serum. Intrathecal IgG- Synthesis according to Reiber quotients diagrams. IgG- Index = CSF/serum IgG:CSF/serum albumin >0.7. Barkhof = 3 of 4 Barkhof criteria [21] fulfilled.

## Discussion

Increasing recognition of the pathogenetic relevance of B lineage cells in MS encouraged the evaluation of B cell-associated biomarker candidates [22], [35]. OCB were shown to be an independent risk factor implementing an almost two-fold increased risk of having a second relapse [9], and we demonstrated the polyspecific intrathecal B cell response against neurotropic viruses MRZR as well as the B-cell attracting chemokine CXCL13 to be of prognostic relevance in CIS [23], [24]. Both OCB and MRZR supposedly reflect a polyspecific B cell response in MS driven by a B cell-enhanced environment in which B lineage cells, notably long-lived plasma cells, can survive for many years, and could differentiate to antibody secreting cells independently of antigen presence in a bystander reaction promoted by T-cells [22], [35].

Ig free light chains (FLCs) are “sideline products” of the Ig synthesis by B lymphocytes [36]. Human Ig molecules contain two identical heavy chains and two identical light chains, which exist as kappa or lambda isotypes, and are linked to heavy chains by covalent bonds [36], [37]. During the production of intact immunoglobulins, plasma cells produce an excess of kappa and lambda light chains that are secreted as FLCs [38]. These FLCs can exist as monomers (22–27 kDa) or dimers (44–55 kDa) [39], and can normally be detected in many biological fluids including serum, urine, synovial fluid, and CSF [40], [41]. While FLCs were originally perceived to be irrelevant bystander products of plasma cell Ig production, they are increasingly recognized to have important functions, including participation in mast cell-driven hypersensitivity, anti-angiogenic and proteolytic activities, and protein targeting [37]. In MS, increased kappa monomer and dimer levels in the CSF were previously reported [14].

Our observation of elevated KFLC in the CSF of patients with MS and pathogen related CNS diseases support previous studies [16], [18], [20], [42], [43] that determined KFLC in CSF of MS patients. However comparability of KFLC levels is low due to differences in methods. Both qualitative methods like IEF with immunoblotting [43] and quantitative methods like radioimmunoassay [16], [44], and nephelometry [18]–[20], [42] were used to determine FLC in CSF.

Due to a significant correlation of CSF KFLC with serum KFLC and CSF-serum ratio of albumin (Q albumin) we introduce reference values of Q KFLC in relation to Q_alb_, which is a widely accepted quantitative measure of blood-CSF barrier function [45].

As a new aspect, we analyse the prognostic relevance of KFLC and other parameters regarding the occurrence of further relapses in a large and clinically well-defined cohort of patients with CIS. Here, we showed OCB to have the highest sensitivity (92.1%) and highest negative predictive value (81.3%) for conversion of CIS to MS as compared to Barkhof criteria (highest specificity (88.2%)). We observed Q KFLC to be nearly as sensitive as CSF OCB in predicting conversion of CIS to MS (Table 3).

The correlation of Q KFLC with CSF leukocyte count, intrathecal IgG synthesis and the B cell attracting chemokine CXCL13 possibly is in line with the polyspecific intrathecal B cell response in MS [23], [24]. Similarly Q KFLC was elevated in CIS patients with presence of OCB and positive MRZR.

So far, OCB are the most widely used CSF test to support the diagnosis of MS and to predict conversion of CIS to MS. OCB s in CSF that are absent in serum indicate intrathecal IgG synthesis. In the classification by Andersson et al 1994 [48] five OCB patterns are described (legend of Figure 4). However, isoelectric focussing for detection of OCB is highly laborious and methodologically demanding. Furthermore, interpretation of OCB is frequently equivocal, especially if banding patterns are weak or single bands occur in the CSF only [13], [46], [47]. Although quality control data from the UK for assessment of OCB s assessed by IEF showed a very high analytical sensitivity and specificity for 114 laboratories participating [25], quality control surveys performed regularly in Germany by INSTAND e.V. (unpublished data) showed that false results can occur due to the different separation and identification techniques used. In contrast, interpretation of KFLC is unequivocal as it provides a quantitative measure. Furthermore, it is methodologically simple, as the measurement can be performed either using ELISA or nephelometry.

The significant correlation of Q KFLC with positive OCB (Figure 3) supports observations from other studies that suggest CSF KFLC to be elevated in patients with intrathecal IgG synthesis [42], [43]. Comparing subgroups of patients with different OCB patterns [48], Q KFLC was significantly elevated in type 2, 3 and 4 as compared to type 1 (Figure 4). However, in contrast to OCB, quantitative KFLC is not sufficient to classify the humoral response according to the number of antibody clones produced. To our knowledge, quantitative KFLC may only indicate presence of intrathecal humoral immunoresponse independent of the type of clonality, i.e. poly-, oligo or monoclonal origin.

In conclusion, our data indicates the relevance of KFLC as a marker of polyspecific IgG response in the CSF of MS and CIS patients. Similar to oligoclonal bands KFLC add information in predicting conversion of patients with CIS to MS. Although the measurement of CSF KFLC is a rapid and quantitative easy to standardize tool, it is almost equal but not superior to OCB with regard to diagnostic sensitivity and specificity in patients with early MS. Additionally, these findings underline the relevance of CSF parameters in MS and CIS. Although CSF analysis is no longer a mandatory part of diagnostic criteria in MS [26], it is essential to exclude other differential diagnoses, and due to its close proximity to the disease pathology in brain and spinal cord, CSF remains an important research tool to understand the pathophysiology of MS [12], [49].
